# Developing elite *Neurospora crassa* strains for cellulosic ethanol production using fungal breeding

**DOI:** 10.1007/s10295-017-1941-0

**Published:** 2017-04-20

**Authors:** Joshua C. Waters, Andrew Nixon, Morgan Dwyer, Justin C. Biffinger, Kwangwon Lee

**Affiliations:** 1 Department of Biology, Rutgers, The State University of New Jersey, Camden, NJ 08103 USA; 2 Center for Computational and Integrative Biology, Rutgers, The State University of New Jersey, Camden, NJ 08103 USA; 3 Chemistry Department, US Naval Research Laboratory, Washington D.C., 20375 USA

**Keywords:** Natural variation, Cellulosic ethanol, Cellulase, Fungal breeding, Strain improvement

## Abstract

The demand for renewable and sustainable energy has generated considerable interest in the conversion of cellulosic biomass into liquid fuels such as ethanol using a filamentous fungus. While attempts have been made to study cellulose metabolism through the use of knock-out mutants, there have been no systematic effort to characterize natural variation for cellulose metabolism in ecotypes adapted to different habitats. Here, we characterized natural variation in saccharification of cellulose and fermentation in 73 ecotypes and 89 laboratory strains of the model fungus *Neurospora crassa*. We observed significant variation in both traits among natural and laboratory generated populations, with some elite strains performing better than the reference strain. In the F1 population N345, 15% of the population outperformed both parents with the top performing strain having 10% improvement in ethanol production. These results suggest that natural alleles can be exploited through fungal breeding for developing elite industrial strains for bioethanol production.

## Introduction

Depletion of global resources and increasing environmental concerns have illustrated the need for the development of renewable and sustainable resources. Cellulosic biomass, the most abundant resource on the planet, is capable of being converted into fuels or chemicals after the plant biopolymers are degraded to fermentable sugars. One of the important bio-products that can be obtained from cellulosic biomass is ethanol for liquid transportation fuels. The successful implementation of scalable bioethanol production as an alternative fuel has been demonstrated in Brazil [19]. However, the recalcitrance (resistance to degradation) of lignocellulose presents a large hurdle toward efficiently utilizing the abundant carbohydrates in plant biomass [6].

Current methods for converting plant biomass to value-added products are expensive and inefficient [29]. Lignocellulose is decomposed to simple sugars through the use of enzyme cocktails derived from industrial fungi, such as *Trichoderma reesei* [29]. The liberated sugars then need to be recovered and fermented to final products by various other species of fungi [1, 2]. These steps are traditionally carried out separately, due to high energy demands for enzyme production and energy limiting conditions of fermentation. An alternative approach, consolidated bioprocessing (CBP), attempts to combine these steps to reduce the overall costs of production, however, there are further technical drawbacks to this process from an engineering standpoint when considering implementation of CBP on an industrial scale and economically viable methods for the recovery of dilute ethanol from industrial scale fermentation cultures. Perhaps the most important limitation is the lack of a single organism that is equipped to efficiently perform all of the requisite processes for CBP; enzyme production, substrate hydrolysis, and fermentation of the liberated hexose and pentose sugars. Before tackling the technical limitations of scalability, it is important to identify organisms that are efficient in all of the requisite physiological processes.

Although yeasts (especially *Saccharomyces*) are the most efficient fermenters of hexose sugars, many are poorly adapted to fermentation of pentose sugars (a large component of plant biomass) and are unequipped for decomposition of cellulose to fermentable sugars [22, 32]. On the other hand, the main fungus used to generate the exoenzymes responsible for decomposition of cellulose to simple sugars, *T. reesei*, is ill-equipped to ferment the sugars it releases from cellulose through the process of saccharification [33]. In attempts to overcome these hurdles researchers have attempted to engineer organisms that are capable of robust cellulase expression and efficient fermentation of both hexose and pentose sugars to consolidate the production stages into one-stage CBP [5, 13, 16, 17, 25]. There are, however, filamentous fungi capable of producing the exoenzymes to decompose cellulosic plant biomass to simple sugars, along with the full sets of enzymes required to ferment both the hexose and pentose sugars of plant biomass to ethanol; among them is the model filamentous fungus *Neurospora crassa* [3, 7, 35, 36].

Although the cellulolytic system of *N. crassa* has been characterized and investigations have been carried out to assess the potential of laboratory strains to decompose a variety of pure and natural substrates, there have been no systematic efforts to characterize the variation within and across populations arising from allelic variation [3, 7, 9, 24, 26]. The genetic malleability, fast growth, and the well-developed tools and protocols for working with *N. crassa* make it a prime organism for studying the combined process of decomposing and fermenting cellulosic substrates simultaneously. In addition to the plethora of available resources and tools, over 2000 *Neurospora* ecotypes are available in the Fungal Genetics Stock Center. We reasoned that these genetic resources are valuable for developing a strain for industrial usage. To achieve this goal, first, we characterized the natural variation in saccharification and fermentation that exists among ecotypes. Second, we generated a population by crossing the two top performing ecotypes to test if we can generate better performing strains in saccharification and fermentation. This process we call ‘fungal breeding’ in this study; developing an elite strain with desirable traits by crossing two related or unrelated parental strains. We have observed a significant variation for both traits in the natural and laboratory populations. Furthermore, a correlation was observed among the ecotypes' abilities to ferment hexose and pentose sugars, suggesting that those ecotypes are highly capable of fermenting hexose sugars and may also be well-equipped for fermenting pentose sugars. Interestingly, there was no correlation observed between the amount of total exoenzyme produced and saccharification capability, suggesting that there are qualitative differences in exoenzyme production that underlie the observed high saccharification potential in some of the elite strains.

## Materials and methods

### Strains, propagation, and crosses

73 natural isolates of *N. crassa* and the wild-type reference strain (FGSC2489) were ordered from the Fungal Genetics Stock Center (FGSC) [11, 21], and grown from long-term stock prior to each experiment on Vogel’s minimal media slants (1× Vogel’s Salts, 2% Sucrose, 1.5% Agar, pH 5.8), whereas a mutant strain carrying a deletion of an extracellular β-glucosidase (⊿Bgl, FGSC18387; NCU08755) from the FGSC was grown up on Horowitz Complete Media (1X complete salts, 1.6% glycerol, 5% casein hydrolysate, 0.5% yeast extract, 0.5% malt extract, 1.5% agar, pH 5.8). *T. reesei* (QM9414) Simmons Anamorph ATCC26921 was ordered from American Type Culture Collection and the freeze-dried pellet was propagated on Potato Dextrose Agar (2% potato dextrose broth, 1.5% agar, pH 5.8) according to the supplier’s instructions. Spore suspensions in High Glucose Liquid Media (HGLM) (1X Vogel’s salts, 2% glucose, 0.5% l-Arginine, pH 5.8) were used to generate mycelial mats in petri plates, which were used to generate replicate mycelial pads for each experiment using a bore punch.

Top performing ecotype strains from saccharification and fermentation screens were each crossed against strains of both mating types (FGSC2489, Mat A and FGSC18387, Mat a) on crossing media slants (1X Westergaard’s salts, 2% Sucrose, 5% Biotin, 1.5% Agar, pH 5.8). Successful crosses were used to type each strain against the mating type it was able to cross with. The two best strains with opposite mating types (JW220, Mat A and JW228, Mat a) were successfully crossed to generate the N345 population. Individual spores were picked, heat-shocked at 60 °C for 30 min, and germinated on minimal media slants. A total of 89 germinated progeny of the cross were studied in this study.

### Plate clearing assay

Plate clearing assays were performed using Congo Red indicator as described by Meddeb-Mouehli et al., in which 4 gauge mycelial pads from spore suspension in HGLM were inoculated onto Carboxymethylcellulose (CMC)/Sorbose agar (1X Vogel’s salts, 0.5% CMC, 2% Sorbose, 1.5% Agar) and plates were flooded 3 days post-inoculation with 0.1% Congo Red for 20 min, and washed sequentially with 1 M NaCl for 15 min [23]. All samples were performed in biological triplicates. Images of the zone of hydrolysis and fungal growth were captured with a Nikon D7000 and analyzed with ImageJ software. Plate clearing assays revealed differential growth and zones of hydrolysis among the natural isolates screened in this study. Despite the addition of sorbose to the medium to restrict lateral growth isolates demonstrated varied responses to sorbose. Therefore, since some strains were able to accumulate more cell mass, and therefore, secrete more protein, an index was created in an attempt to normalize enzyme secretion to the amount of cell mass increase. A cell mass increase (CMI) index was created to determine how much cell mass was accumulated during growth on the cellulose substrate. However, it is important to note that measuring lateral growth does not account for how dense the cell mass may be, and is, therefore, not a precise measurement of the total increase in cell mass. Nevertheless, it was considered useful in assessing how much of the consumed substrate was utilized for growth. A Cellulase Production Index (CPI) was created in an attempt to normalize the area of hydrolysis to the total cell mass capable of producing enzyme. Finally, a Substrate Utilization Index (SUI) was created as an attempt to score individuals based on their cellulase production relative to how much hydrolyzed substrate was diverted toward growth, with high scores representing strains with large areas of hydrolysis and little utilization of substrate for growth. Individuals with high CPI are of interest for their ability to hydrolyze more substrate with less cell mass required, while individuals with high SUI are of interest for ethanol production since less of the hydrolyzed substrate is deferred to growth and is available for fermentation.

The indices were calculated as follows: Area of Cellulase Activity (ACA) = Area of clearing; Cell Mass Increase (CMI) = area of growth _final_ − area of pad _initial_; Cellulase Production Index (CPI) = ACA/area of growth _final_. Substrate Utilization Index (SUI) = CPI/CMI.

### Enzyme activity assays

A modified FPA assay was performed using 96-well plates as described by Camassola and Dillon, in which secreted protein extracts were taken from wells in 6-well plates containing 1% CMC broth (1X Vogel’s salts, 1% CMC) 4 days after inoculation with 10 gauge mycelial pads [4]. Culture broth containing secreted enzyme was filtered and centrifuged at 13.2 k rpm to remove any fungal cells or debris. 50 μL of supernatant was added to 100 μL of 50 mM sodium acetate buffer pH 5.6 in a 96-well deep-well plate, which was then equilibrated to 50 °C for 5 min in a hot-water bath. A 5 mg strip of Whatman Grade No. 1 filter paper was submerged in the solution, and the plate was incubated at 50 °C for 60 min. After 60 min, 300 μL of DNS Reagent was added to stop the enzymatic reaction and visualize glucose equivalents. The plate was incubated at 100 °C for 10 min to develop color, then transferred to an ice bath to stop color development. 100 μL of Enzyme/DNS mixture was transferred to a clear-bottom assay plate, diluted with 200 μL of diH2O, and absorbance was measured at 545 nm. The concentration of reducing equivalents released was determined with standard curves generated with glucose standards. All samples were performed in biological triplicate. Due to the relatively low concentration of secreted enzymes, activity could not be quantified in conventional filter paper units, which requires the production of at least 2 mg/ml of glucose equivalents, therefore, results were presented in the form of the concentration of reducing equivalents released by each aliquot. Similarly, a modified carboxymethylcellulase (CMCase) assay was performed using soluble 2% CMC as the reaction substrate instead of Whatman filter paper. The enzymatic reaction was carried out for 30 min instead of 60 min, however, the rest of the procedure was carried out as it was in the FPA assay.

### Fermentation

To characterize fermentation among the top ecotypes (JW-16, JW-60, JW-160, JW-161, JW-176, JW-220, JW-228, JW-234) based on their performance in preliminary experiments investigating cellulolytic potential, the top nine candidates, along with wild-type *N. crassa* strain (FGSC2489), and commercial brewer’s yeast were screened for variation in fermentation of glucose. Mycelial mats of *Neurospora* were generated from spore suspensions in HGLM. Mycelial pads were punched from the mats with a 10 gauge punch, rinsed in sterile deionized water, and inoculated into 15 ml conical tubes containing 15 ml of 2% Glucose, 2% Xylose, or 1% CMC. 500 μl of yeast suspension was inoculated into 15 ml of corresponding substrate as positive control for glucose fermentation. The tubes were tightly sealed with screw-on caps to ensure anaerobic conditions. Cultures were incubated at room temp for 9 days, after which they were centrifuged at 2500 rpm for 10 min. at 4 °C. The supernatant was recovered and filtered through grade No. 1 Whatman filter paper, centrifuged at 5000 rpm for 5 min and the supernatant was recovered for analysis. All samples were performed in biological quadruplicate. The recovered supernatant was aliquoted into HPLC vials for ethanol analysis by HPLC. HPLC quantitation was performed using a Varian ProStar HPLC with a Varian ProStar Autosampler and a Varian 356-LC Refractive Index Detector. An isocratic elution was used with an Agilent Hi-Plex H^+^ (300 mm × 7.7 mm ion-exchange column) with 5 mM H_2_SO_4_ at a flow rate of 0.7 ml/min at 60 °C. The concentration of ethanol present was determined from a standard curve based on ethanol standards with a known concentration.

A 96-well format for fermentation was carried out in deep-well plates to characterize the amount of fermentation among the lab generated N345 first filial (F1) generation. Replicates for each strain were collected from mycelial mats with a six gauge punch and inoculated into 750 µl of HGLM (2% glucose), sealed with aluminum ThermowellTM seals, and allowed to ferment for 7 days in 12:12 LD conditions at 25 °C. All samples were performed in biological quadruplicate. After fermentation, 600 µl of media was recovered and cell debris was removed by sequential centrifugation at 13.2 k rpm for 5 min. Recovered supernatant was analyzed at NRL as previously described.

Finally, to characterize direct fermentation of cellulosic biomass, six gauge mycelial pads of FGSC2489, JW220 and JW228 (cross parents), and N345-2 (best cross progeny) were inoculated into 750 µl of 2% Miscanthus broth and incubated at ambient room temperature for 7 days. Culture broth was recovered and sequentially centrifuged at 13.2 k rpm to remove fungal debris and residual plant matter. Supernatants were transferred to HPLC vials and analyzed for ethanol content as previously described.

### Statistical analysis

T-tests and Single Factor ANOVA were performed in Microsoft Excel using the data analysis tool-pack. Prism Graph Pad was used to test for Pearson correlations and for construction of graphs.

## Results

### Natural variation in saccharification and fermentation among *Neurospora* ecotypes

**Fig. 1:**
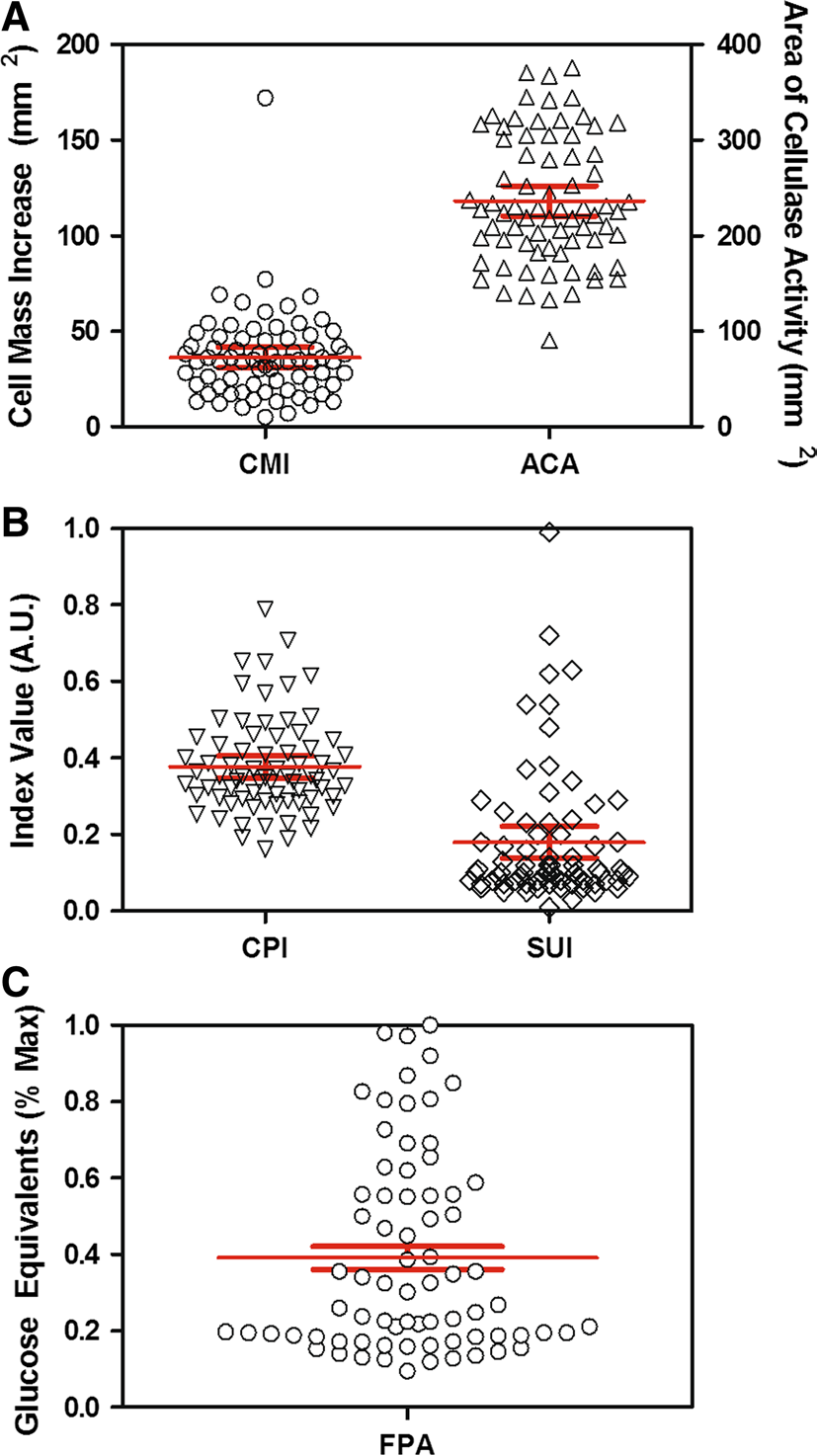
Natural variation in cellulolytic activity among natural isolates. **a**, **b** Significant variation was observed among 73 natural strains in the indices analyzed in plate clearing experiments. The area of cellulase activity (ACA), cell mass increase (CMI), and cellulase production index (CPI) demonstrated normal distributions, while substrate utilization index (SUI) was positively skewed. The skew of SUI is expected to arise from variation in sorbose resistance opposed to cellulolytic activity, as sorbose was used to restrict lateral growth of hyphae. **c** Significant variation was observed in FPA assay among natural strains, presented as percentage of glucose equivalents released by top performer since values fall outside the range of the standard curve used. *Red bars* represent mean and 95% CI

To quantitatively measure the level of cellulase expression by different ecotypes of *Neurospora*, we have used several indices, cell mass increase (CMI), area of cellulase activity (ACA), and Cellulase Production Index (CPI) (“Materials and methods”). We reasoned that a desirable strain for ethanol production is the strain that can hydrolyze the largest amounts of available cellulose without diverting too much of the liberated sugars toward its own cellular growth. Thus, Substrate Utilization Index (SUI) was defined as the ratio of CPI to CMI, with larger values indicative of high levels of hydrolysis with minimal growth. In general, the observed data for ACA, CMI, and CPI appeared to be normally distributed, however, the data for SUI shows a strong positive skew (Fig. 1a, b).

**Fig. 2:**
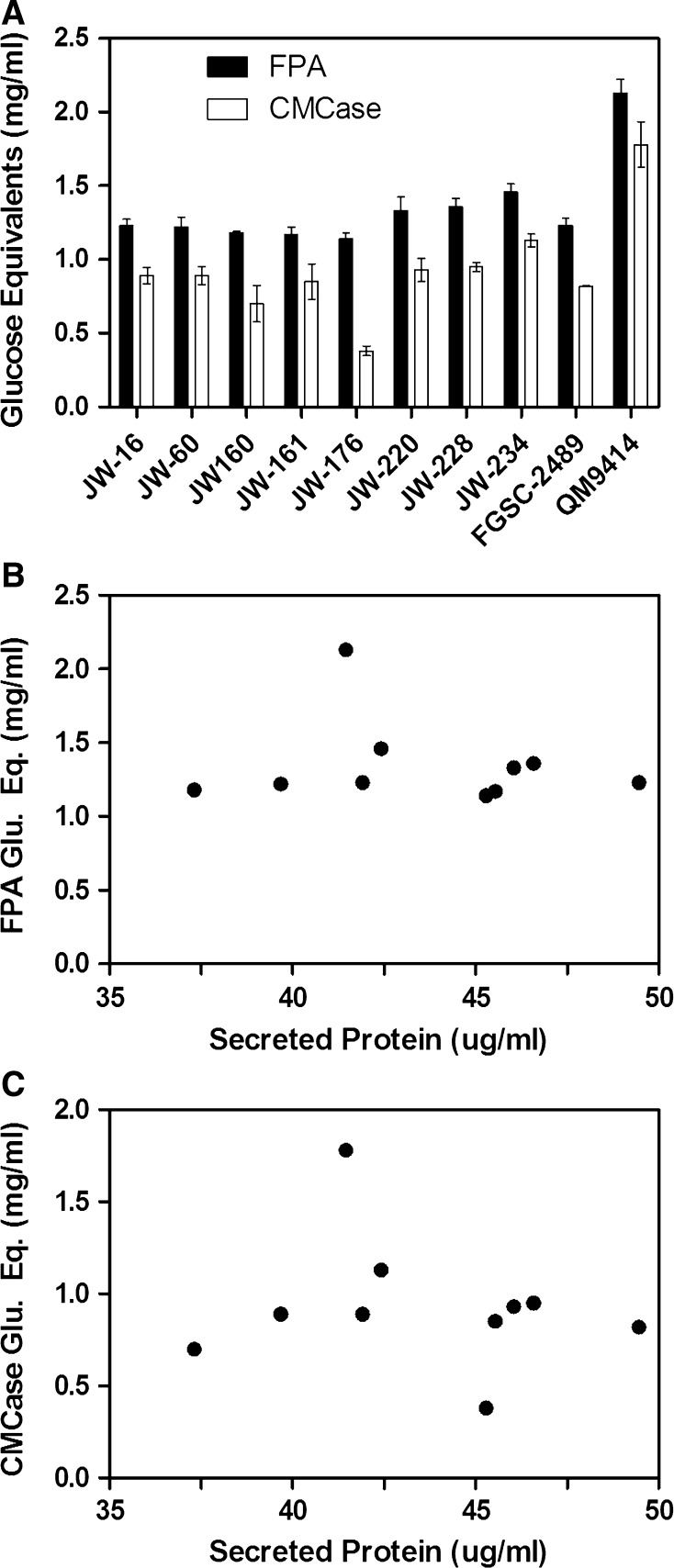
Natural variation in cellulolytic activity among top performing natural ecotypes. Variation was observed among the top performing strains activity in the FPA assay and CMCase assay (**a**). No correlation was observed between the amount of protein secreted and the level of cellulase activity measured by FPA assay (**b**) or CMCase assay (**b**) (*R* ^2^ = 0.02285, *p* = 0.6768 and *R* ^2^ = 0.03114, *p* = 0.6258, respectively). QM9414 (**a**) is *T. reesei*, used as a standard for cellulase activity. *Error bar* represents one standard deviation

The activity of secreted enzyme from each isolate was determined using a modified filter paper activity **(**FPA) assay as previously described (“Materials and methods” and [4]). An almost tenfold difference was observed among the activity of the natural isolates tested (Fig. 1c). The top performing strains from preliminary FPA screens were selected for comparison based on their cellulolytic enzyme production. Glucose equivalents released during FPA and CMCase assays among the top ten strains showed minor variation (Fig. 2a) with QM9414 generating almost twice the concentration of glucose/mL compared to the rest of the strains tested in the current study. There was no correlation between secreted protein and cellulolytic activity of the top performing strains in either assay, suggesting that the key catalytic enzymes for degradation of cellulose, cellobiohydrolases and endo-glucanases, represent only a small fraction of secreted protein (Fig. 2b, c). All strains secreted proteins to a concentration of 35–50 μg/ml in the course of the experiments, resulting in approximately 1.2 ± 0.2 mg/ml of glucose except for QM9414 which produced twice the concentration of glucose.

**Fig. 3:**
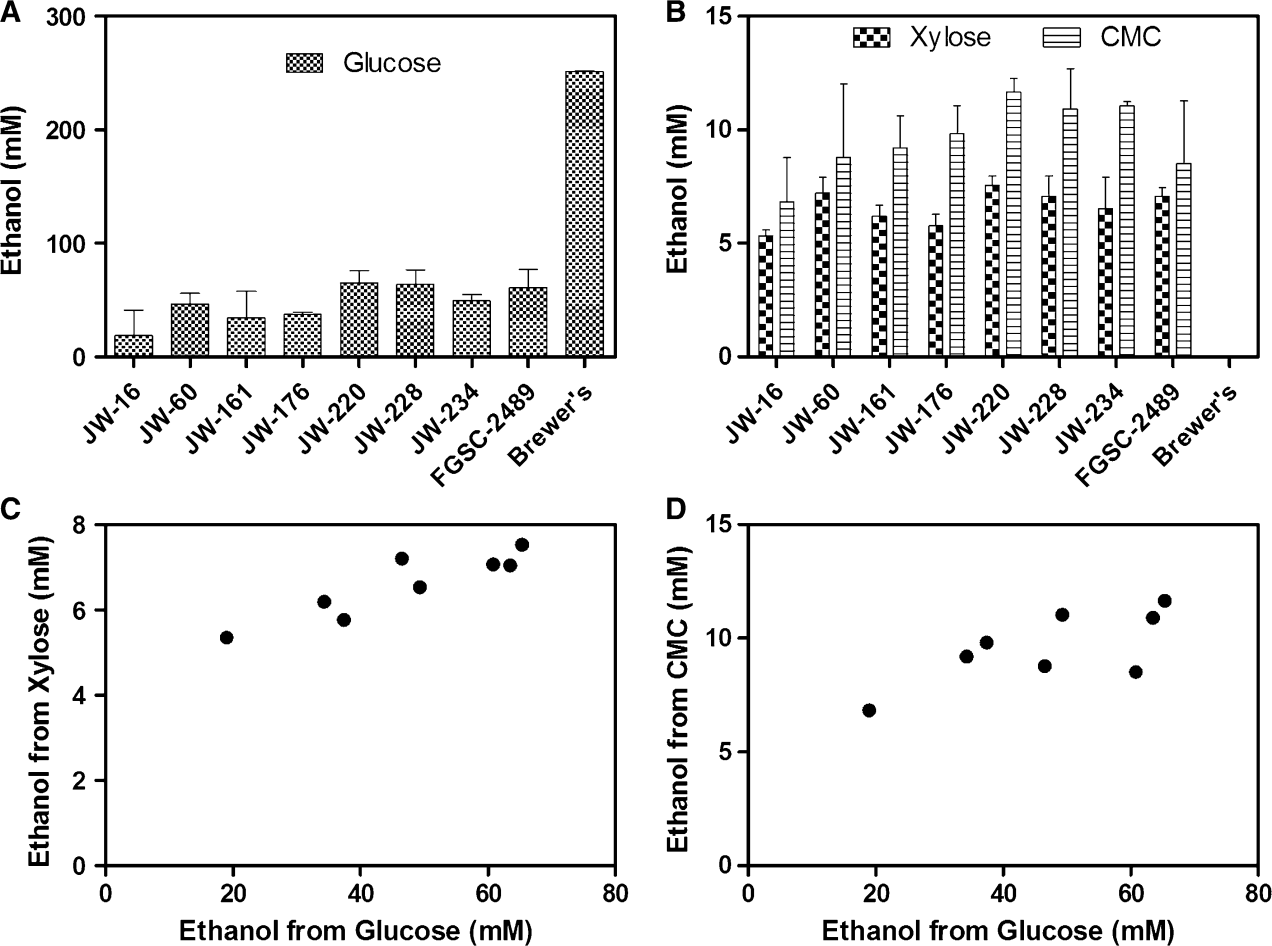
Natural variation in fermentation among top performing ecotypes. **a** Ethanol produced from fermentation of glucose. **b** Ethanol produced from fermentation of 2% Xylose or 2% CMC. **c**, **d** Correlations were observed between ethanol produced from fermentation of glucose and xylose (**c**), and between fermentation of glucose and CMC (**d**) (*R* ^2^ = 0.7511, *p* = 0.0025 and *R* ^2^ = 0.6711, *p* = 0.0069, respectively). *Error bars* represent one standard deviation

These same strains were then compared based on the concentration of ethanol that resulted from the fermentation of glucose, xylose, and CMC as substrates. The top performing strains from the combined cellulase production screens were selected for this analysis. An approximately sixfold difference was observed among the selected strains for fermentation of glucose, however, commercial brewer’s yeast proved superior (Fig. 3a). There was no significant difference between the same strains when fermenting the pentose sugar xylose, while a twofold difference was observed for the selected strains when fermenting the more complex polysaccharide CMC (Fig. 3b). Brewer’s yeast was unable to ferment xylose or CMC since it is lacking the enzymes for conversion of xylose to fermentable substrates and hydrolysis of cellulose to fermentable glucose (Fig. 3b). A correlation was observed between fermentation of glucose and xylose among strains (Fig. 3c), while a weaker correlation was observed between fermentation of glucose and CMC (*R* ^2^ = 0.7511, *p* = 0.0025 and *R* ^2^ = 0.6711, *p* = 0.0069, respectively) (Fig. 3d).

### N345 cross population

**Fig. 4:**
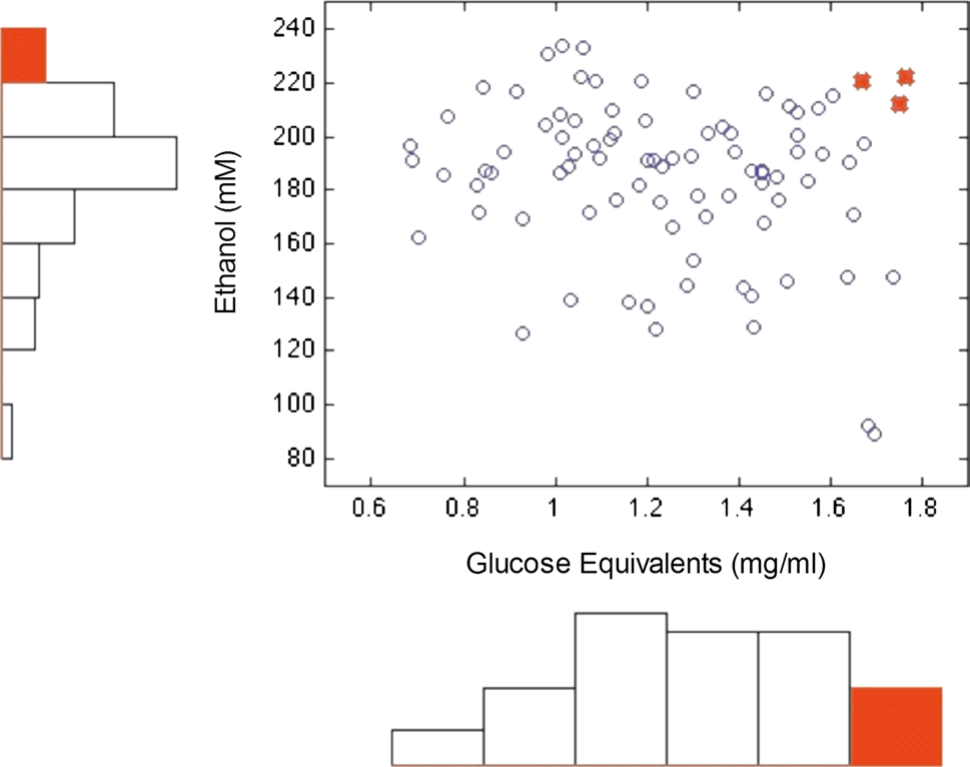
Natural variation in cellulolytic activity and fermentation among a laboratory population (N345 population). A 2-dimensional scatterhist plot illustrating distributions for saccharification of cellulose (*x* axis) and fermentation of glucose (*y* axis). *Red dots* represent strains with the highest potential for both traits

To explore the potential of fungal breeding as an approach to enrich cellulase production and fermentation, the top performing natural strains of opposite mating types were crossed and the progeny were tested for saccharification of cellulose and fermentation of glucose. Although significant variation was observed in the FPA assay, only one strain (N345-2) was able to outperform both parents, and only four strains were able to outperform the lesser parent (ANOVA *p* value = 1.67 × 10^−22^) (Fig. 4). We also sought to assess the potential of fungal breeding as a route to enrich fermentation. Therefore, the progeny obtained from crossing the top performing parents were also screened for fermentation of glucose. Significant variation was observed among the 87 progeny tested (ANOVA *p* value = 5.15 × 10^−7^). Importantly, 13 of the progeny were able to outperform both parents demonstrating the ability of fungal breeding to enrich fermentation capacity in a single generation.

### Biomass fermentation

**Fig. 5:**
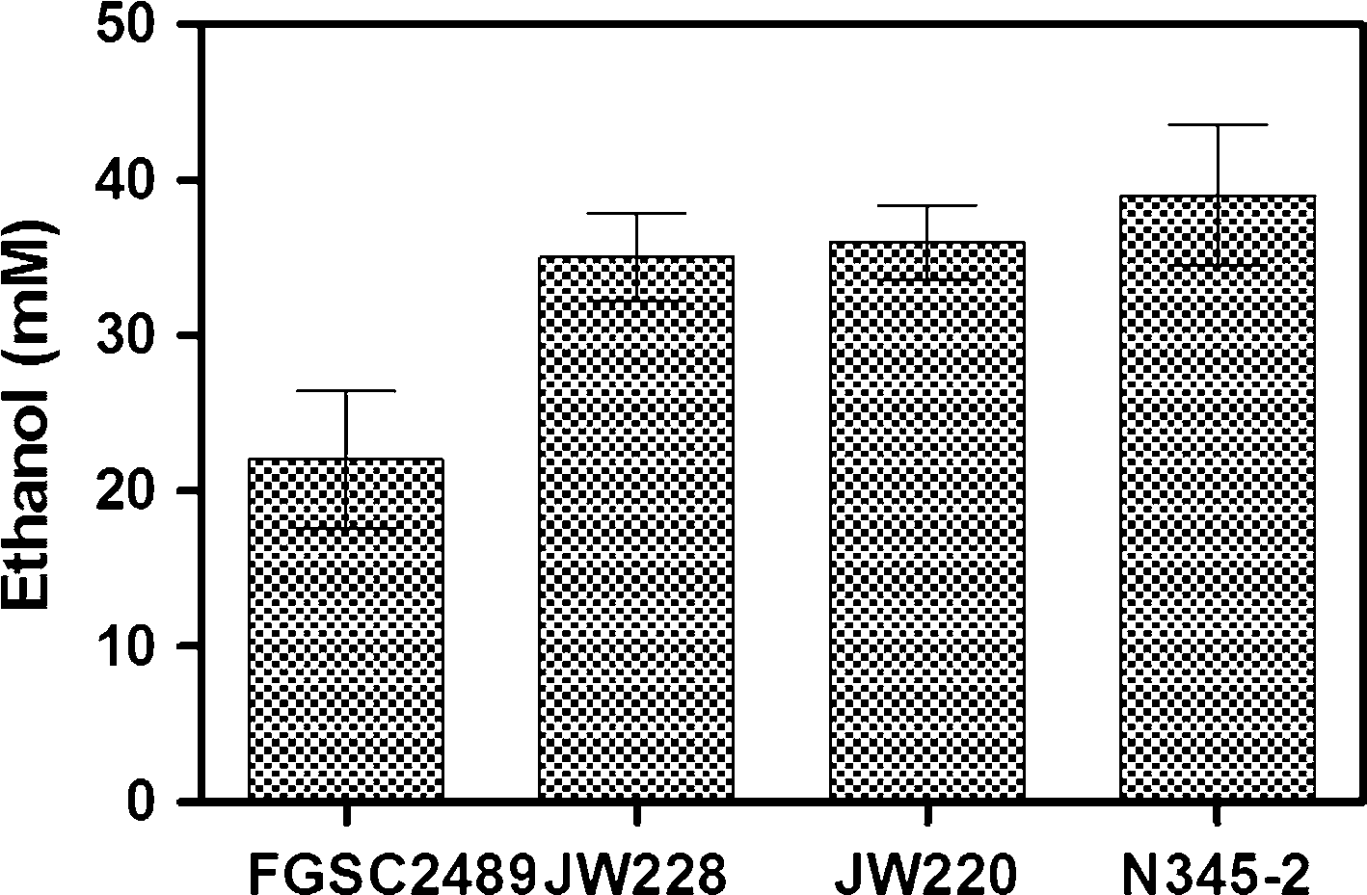
Fermentation of a High Energy crop by elite ascensions. Elite ascensions (JW220, JW228, and N345-2) were able to produce more ethanol from a 2% Miscanthus culture than the sequence strain (FGSC2489) which serves as a wild-type reference. Significant difference was observed between each strain and reference strain using 2-tailed *t* test (JW220 *p* = 0.000005, JW228 *p* = 0.000005, N345-2 *p* = .00007). *Error bars* represent one standard deviation

Finally, to validate our hypothesis that strains capable of decomposing pure defined cellulose substrates should also be able to decompose more complex natural substrates, fermentations were performed with FGSC2489 and elite ecotypes JW220, JW228, and N345-2 using the high-energy crop Miscanthus as the sole carbon source. While all stains were able to directly ferment lignocellulosic biomass to ethanol, the elite ascensions chosen from preliminary screenings were able to produce almost twice as much ethanol as the reference strain (Fig. 5). This result supports the hypothesis that elite ecotypes exist that are better adapted for direct fermentation of lignocellulosic substrates, and that natural variation could be exploited through selective fungal breeding or genetic engineering to enrich for traits involved in cellulosic biofuel production.

## Discussion

Various attempts have been made to reduce costs and labor of cellulosic ethanol production via CBP, however, these attempts have utilized organisms engineered to incorporate cellulolytic or fermentation pathways from other species [10, 14, 16, 17, 25]. While investigations aimed at understanding cellulolytic and fermentative systems, and even attempts at CBP have been made utilizing wild-type and gene deletion mutants of *N. crassa*, none have investigated natural variation due to allelic effects as a source of elite strains for CBP [8, 18, 27, 30, 31]. Here, we investigated variation in saccharification of cellulose and fermentation among natural and laboratory populations using model cellulose substrates to identify elite strains for CBP. Although model cellulose substrates, such as CMC and Whatman filter paper, are not as complex as natural cellulosic substrates, they have proven to be effective tools for screening endoglucanase and total cellulase activity, respectively [4, 12, 15, 20, 28, 34].

Significant variations in cellulase secretion (Figs. 1, 2) and in fermentation (Fig. 3) were observed among natural strains adapted to different habitats, as well as the lab generated N345 population (Fig. 4). The lack of correlation between total exoenzymes production and enzyme activity suggests that qualitative differences in the enzymes produced may explain the observed variation. Whether the observed differences in cellulase activity are the result of differential expression of cellulolytic enzymes among strains or differences in relative abundances of cellulase enzymes present remains to be determined. In the progeny, we could identify multiple strains with increased fermentation capacity, however, only one strain was able to outperform the parents in cellulase activity assay and fermentation (Fig. 4). Future studies should investigate the richness of enzyme production and how enzyme diversity affects saccharification.

It was particularly interesting that we have observed a significant variation in fermentation, with a fivefold difference in fermentation of glucose among ecotypes (Fig. 3a). Although there was minimal variation in fermentation of xylose, there was a correlation in general fermentation ability, with strains more adept at fermenting glucose also producing more ethanol from xylose (Fig. 3c). There was also a weaker correlation observed between fermentation of simple sugars and fermentation of cellulose (Fig. 3d). This is likely the result of the underlying, rate-limiting step of saccharification, which also varied among strains. Therefore, the potential for developing an industrial fungal strain for ethanol production from plant polysaccharides lies in exploring the elite alleles in the ecotypes responsible for converting complex sugars to monosaccharides, and their fermentation to ethanol. As a proof of the concept, we found that there exists almost twofold difference in ethanol production from the natural ecotypes (JW228, JW220, and N345-2) over the reference strain (FGSC2489) (Fig. 5).

## Conclusion

The successful enrichment of fermentation ability in the first F1 generation from a cross between top performing ecotypes illustrates the potential for fungal breeding to generate elite strains with improved traits for industrial purposes. Further investigation into the molecular mechanisms underlying variation should be able to identify specific allelic combinations for further strain optimization through breeding and genome-wide metabolic engineering. We illustrate here that *N. crassa* is a strong candidate for consolidated bioprocessing of biomass to ethanol. In addition to the previous efforts in screening knock-out strains to understand a transcriptional response in cellulose metabolism, characterizing allelic effects in ecotypes will provide novel insights in designing industrial *N. crassa* strains.
